# Silicon Wafer-Based Platinum Microelectrode Array Biosensor for Near Real-Time Measurement of Glutamate *in Vivo*

**DOI:** 10.3390/s8085023

**Published:** 2008-08-27

**Authors:** Kate M. Wassum, Vanessa M. Tolosa, Jianjun Wang, Eric Walker, Harold G. Monbouquette, Nigel T. Maidment

**Affiliations:** 1 Dept. Psychiatry & Biobehavioral Sciences, Semel Institute for Neuroscience and Human Behavior, UCLA, Los Angeles, CA, USA; E-mails: kwassum@ucla.edu (K.M.W.); ewalker41@gmail.com (E.W.); 2 Chemical and Biomolecular Engineering Dept., UCLA, Los Angeles, CA, USA; E-Mails: vtolosa@ucla.edu (V.M.T.); wangjj@ucla.edu (J.W.)

**Keywords:** Glutamate Biosensor, Constant Potential Amperometry, Nafion, Polypyrrole, Central Nervous System

## Abstract

Using Micro-Electro-Mechanical-Systems (MEMS) technologies, we have developed silicon wafer-based platinum microelectrode arrays (MEAs) modified with glutamate oxidase (GluOx) for electroenzymatic detection of glutamate *in vivo*. These MEAs were designed to have optimal spatial resolution for *in vivo* recordings. Selective detection of glutamate in the presence of the electroactive interferents, dopamine and ascorbic acid, was attained by deposition of polypyrrole and Nafion. The sensors responded to glutamate with a limit of detection under 1μM and a sub-1-second response time in solution. In addition to extensive *in vitro* characterization, the utility of these MEA glutamate biosensors was also established *in vivo*. In the anesthetized rat, these MEA glutamate biosensors were used for detection of cortically-evoked glutamate release in the ventral striatum. The MEA biosensors also were applied to the detection of stress-induced glutamate release in the dorsal striatum of the freely-moving rat.

## 1. Introduction

The amino acid L-glutamate (glutamate) is the major excitatory neurotransmitter in the mammalian central nervous system and as such underlies not only normal, but also many abnormal behaviors apparent in neurological and psychiatric disorders [1-5]. Therefore, a tool for measuring glutamatergic transmission in a behaviorally relevant manner will greatly aid our understanding of these processes.

A variety of sampling methods for the measurement of extracellular brain chemicals, including glutamate, are available. One commonly used method, microdialysis coupled with high performance liquid chromatography, allows for the selective measurement of many different neuromodulators. Unfortunately, even advanced microdialysis techniques do not offer the temporal resolution required for sophisticated behavioral studies [6]. Behavior, especially motivated behavior, can change within seconds of stimuli presentation [7], and the 5-10 min temporal resolution of microdialysis [6] time-averages these fast changes [7-10]. Electrochemical sensors used with voltammetric recording techniques offer an alternative method for measurement of electroactive neurotransmitters, such as dopamine (DA), with improved temporal and spatial resolution [10]. The non-electroactive nature of glutamate poses difficulties to its sensitive and selective measurement with such techniques. Fortunately, implantable biosensors, analytical tools consisting of both a biochemical recognition element and a physical transducer, circumvent these obstacles.

Amperometric electroenzymatic methods for the near real-time detection of glutamate have been developed using platinum electrodes modified with glutamate oxidase (GluOx) [11-13]. GluOx is a flavoenzyme that catalyzes the oxidative deamination of glutamate in the presence of water and oxygen with the formation of α-ketoglutarate, ammonia and hydrogen peroxide (H_2_O_2_) [14]. Electrooxidation of the enzymatically generated H_2_O_2_ allows for effective glutamate detection [11]. Unfortunately, efficient oxidation of H_2_O_2_ requires a high anodic potential at which electroactive interferents, such as DA and ascorbic acid (AA), are also oxidized and thereby contribute an undesired amperometric current signal [15]. Several approaches have been taken to eliminate electroactive interference, such as immobilization of ascorbate oxidase [16], coating with permselective polymers [15-17], self-referencing [18] and co-immobilization of peroxidase with a redox polymer [19].

In addition to electroactive interference exclusion and temporal resolution, precise spatial resolution is also important to permit measurement of glutamate from discrete brain regions *in vivo*. Glutamate can play differing roles in behavior based on the specific brain region or even subregion in which it is released [20], therefore an optimal biosensor for glutamate would be able to make glutamate recordings from a population of cells within a single brain subregion. Previously, we and others described effective platinum wire-based electrodes for amperometric detection of glutamate [13, 15]. However, the signal-to-noise characteristics and required exposed surface of these electrodes make them sub-optimal for spatially precise glutamate measurements *in vivo*. More recently, we adapted an over-oxidized polypyrrole coating approach to commercially available ceramic MEAs developed by Gerhardt and colleagues (Quanteon, LLC). Here, we describe the fabrication of significantly smaller, silicon wafer-based microelectrode array (MEA) probes coated with both Nafion and polypyrrole (PPy), which reduce signal from the interferents, AA and DA, respectively, to below baseline noise levels while maintaining the fast response time necessary for temporally precise measurements of glutamate *in vivo*. These silicon wafer-based glutamate biosensors have been tested extensively *in vitro* and have been applied to measurement of cortical electrical stimulation- and behaviorally-evoked glutamate release *in vivo*.

## 2. Experimental Section

### 2.1. Reagents

Nafion (5 wt.% solution in lower aliphatic alcohols/H_2_O mix), bovine serum albumin (BSA, min 96%), glutaraldehyde (25% in water), pyrrole (98%), L-glutamic acid, L-ascorbic acid, 3-hydroxy-tyramine (dopamine) were purchased from Aldrich Chemical Co. (Milwaukee, WI, USA). GluOx from *Streptomyces* Sp. X119-6, with a rated activity of 24.9 units per mg protein (U mg^-1^, Lowry's method), produced by Yamasa Corporation (Chiba, Japan), was purchased from Associates of Cape Cod, Inc. (Seikagaku America, MA, USA). Phosphate buffered saline (PBS) was composed of 50 mM Na_2_HPO_4_ with 100 mM NaCl (pH 7.4). Ultrapure water generated using a Millipore Milli-Q Water System was used for preparation of all solutions used in this work.

### 2.2. Instrumentation

Electrochemical preparation of the sensors was performed using a Versatile Multichannel Potentiostat (model VMP3) equipped with the ‘p’ low current option and low current N' stat box (Bio-Logic USA, LLC, Knoxville, TN, USA). *In vitro* and *in vivo* experiments were conducted with a multichannel FAST-16 potentiostat (Quanteon, LLC, Lexington, KY, USA). Electropolymerization of PPy was conducted using a standard three-electrode system, consisting of a platinum wire auxiliary electrode, a glass encased Ag/AgCl in 3M NaCl solution reference electrode (Bioanalytical Systems, Inc., West Lafayette, IN, USA), and a platinum working electrode on our MEA probes. *In vitro* and *in vivo* measurements were conducted using a two-electrode system, with reference electrodes consisting of a glass-enclosed Ag/AgCl wire in 3 M NaCl solution (Bioanalytical Systems, Inc., West Lafayette, IN, USA) or a 200 μm diameter Ag/AgCl wire, respectively. All potentials are reported versus the Ag/AgCl reference electrode.

### 2.3. Electrode Fabrication and Polymer Modification

**Figure 1.**
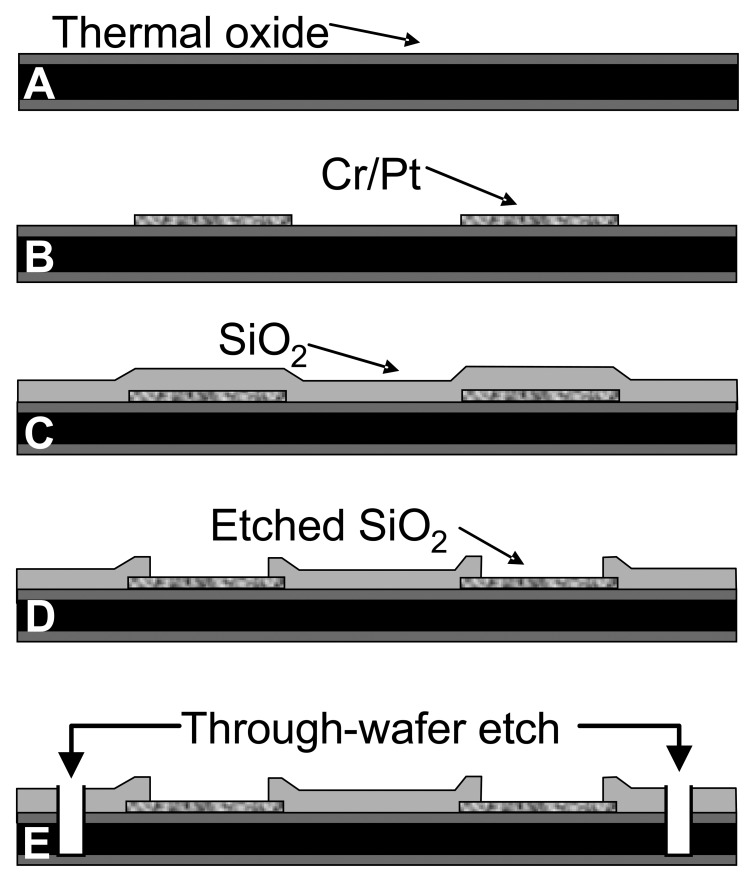
Fabrication process flow diagram of silicon wafer-based MEA probe (cross-section view) **(a)** 1μm SiO_2_ was grown thermally on a 150-μm Si wafer. **(b)** Cr and Pt were deposited by e-beam evaporation followed by a lift-off process to form the electrodes and connections. **(c)** SiO_2_ (1 μm) was deposited as the insulating layer by PECVD. **(d)** The SiO_2_ passivation layer was plasma-etched by RIE at the electrode sites and contact pads. **(e)** A sequential RIE and DRIE through-wafer etch was done to release the probes.

The MEA probes were fabricated at the Nanoelectronics Research Facility at UCLA. A 1 μm thick layer of silicon dioxide was grown thermally on a thin (150 μm) silicon substrate (Figure 1A). The thermal oxide is a high quality dielectric film that electrically isolates the substrate from the metal layer subsequently deposited. Electron-beam evaporation was used to deposit 1000 Å of platinum on a 200 Å chromium adhesion layer. The metal was patterned by photolithography and lift-off to define the bonding pads, connections, and electrode sites (Figure 1B). Next, plasma enhanced chemical vapor deposition (PECVD) was used to deposit a 1 μm layer of silicon dioxide (Figure 1C). This second dielectric layer chemically isolates the connections from solution during electrochemical testing. After patterning of the oxide layer with a conventional photolithographic technique, the contact pads and electrode sites were plasma etched by reactive ion etching (RIE) (Figure 1D). A third photolithographic treatment was performed to pattern the outline of the probes. RIE was then used to etch through the first and second dielectric layers, and deep reactive ion etching (DRIE) by the Bosch process was used to etch through the silicon substrate (Figure 1E).

After the MEA probes were individually released from the wafer they were packaged and chemically cleaned to prepare the electrode surfaces for chemical modification with polymers and enzyme. Packaging involved soldering 28-gauge wire to the platinum bonding pads at the top of the MEA. Each MEA was cleaned with a 1:4 H_2_O_2_:H_2_SO_4_ solution. The tip of the MEA was lowered into the cleaning solution for 3 min and then rinsed with stirred purified H_2_O for 3 min; this process was repeated 3 times. Following cleaning, the electrodes were dried with argon. Each electrode was coated with PPy and Nafion. PPy was electrodeposited by holding the voltage constant at 0.85 V for 2.5–5 min until a total charge density of 20 mC/cm^2^ was reached in a 200 mM argon-purged solution of pyrrole in PBS at pH 7.4. The polymer Nafion was deposited on the sites by rapid dip-coating of the probe tips in the Nafion solution and oven-casting at 180 °C for 4 min, followed by 4 min cooling in ambient air. This process was repeated 3 times. After the polymer treatments, enzyme immobilization was accomplished by chemical crosslinking using a solution consisting of GluOx (2 wt%), BSA (2 wt%) and glutaraldehyde (0.125%). A ∼1 μL drop of the solution was formed on a syringe tip and fixed in place under a microscope. The probe was attached to a micromanipulator (Sutter Instruments) and positioned vertically relative to the enzyme solution droplet. With the aid of the microscope, the MEA was lowered into the enzyme droplet to either coat only the bottom 2, or all 4, electrodes. This was repeated 4 times with each application consisting of 2-3 dips. MEAs coated with PPy/Nafion and GluOx are referred to as MEA glutamate biosensors. The MEAs were sealed in a container with desiccant and stored at 4°C.

### 2.4. Electrode Characterization and Data Analysis

MEA biosensors prepared for glutamate detection were calibrated *in vitro* to test for sensitivity, selectivity and response time to glutamate. *In vitro* testing was carried out using constant potential amperometry with the FAST-16 electrochemistry system. A constant potential of 0.7 V was applied to the working electrodes against a Ag/AgCl reference electrode in 40 mL of stirred PBS at pH 7.4 and 37 °C within a Faraday cage. Data were collected at 80 kHz and averaged over 1 s intervals. After the current detected at the electrodes equilibrated to baseline (approx. 30 min), three 40 μL aliquots of glutamate (20 mM) were added to the beaker to reach a final glutamate concentration of 20, 40 and 60 μM glutamate. Additionally, aliquots of the potential interferents, AA (250μM final concentration) and DA (5-10μM final concentration), were added to the beaker in most tests to determine selectivity for glutamate. In some cases, lower concentrations of glutamate were added to the beaker (5-10 μM final concentration) to more accurately determine glutamate sensitivity. A calibration factor based on analysis of these data was calculated for each electrode on the MEAs to be used for *in vivo* experiments. In order to assess the sensitivity and response time to peroxide at sites uncoated with enzyme aliquots of H_2_O_2_ were also added to the beaker.

Estimations of MEA response time to glutamate were also made *in vitro* using a custom-made flow cell chamber modeled after Lu *et al.* [21]. The MEA was lowered into the plexiglass chamber such that the tip of the probe entered a narrow channel (2 mm diameter × 5 mm depth) through which PBS entered the chamber from below. Using a 60 mL syringe driven by a syringe pump, PBS was infused through the channel at a rate of 4 mL/min. For these experiments, a potential of 0.7 V versus Ag/AgCl was applied across the electrodes, data were collected at 80 kHz and averaged over 0.1 s intervals. A 1 mL sample loop filled with the analyte of interest (glutamate or H_2_O_2_) was used to inject the analyte into the chamber over a 10-15 s period. A computer-directed pneumatic actuator controlled the switching of the sample injector, and the entire setup was housed in an incubator to maintain the temperature at 37 °C. Concentrations of glutamate and H_2_O_2_ ranging from 10-100 μM were used to determine MEA response time. Additionally the flow cell apparatus was used to confirm lack of DA interference at higher concentrations (20 μM). The FAST-16 system allowed precise marking of the event time for each sample injection. Data were output as current as a function of time and analyzed in Microsoft Excel. The response time for H_2_O_2_ detection at bare platinum sites was used as an estimate of the dead time in the system and subtracted from all measurements of response time at coated sites.

### 2.5. In Vivo Electrode Characterization and Data Analysis

The MEA glutamate biosensors were tested in 2 *in vivo* applications. Male Sprague Dawely rats (Charles River) were individually housed on a 12:12 light/dark cycle with *ad libitum* access to food and water. All experimental procedures and surgeries were conducted in accordance with the Institutional Animal Care and Use Committee and UCLA. Standard stereotaxic surgical techniques under halothane anesthesia were used to unilaterally implant a microbiosensor, pre-calibrated to glutamate (see above) into the nucleus accumbens core (NAc) of the ventral striatum (VS) using the following coordinates according to the atlas of Paxinos and Watson (4^th^ ed.) (AP: +1.7, ML: -1.5, V - 6.0) Additionally, a bipolar stimulating electrode (Plastics One, Roanoke, VA) was unilaterally implanted into medial prefrontal cortex (mPFC) (AP: +3.2, ML: -0.8, V -4.4). A 200 μm diameter Ag/AgCl reference electrode was implanted contralaterally. The entire experiment was conducted inside a Faraday cage. The biosensor was connected to the FAST-16 potentiostat and a potential of 0.7 V versus Ag/AgCl was applied. Amperometric data were collected at 80 kHz and averaged over 0.1 s intervals. The electrode signal was allowed to equilibrate to baseline for approximately 30 min prior to application of 0.5 s stimulation trains of 1 ms duration 500-800 μA biphasic square wave pulses at 500 Hz (Coulbourn Instruments, Whitehall, PA) to the mPFC to elicit current changes detected in the VS at the PPy/Nafion/GluOx-coated electrode. Stimulations were administered 30 s apart. Using the Quanteon FAST-16 recording system, a record of the precise timing of the stimulation was included with the electrochemical dataset. An *in vitro* calibration factor was used to convert current changes detected at the electrode into glutamate concentration changes (see above). In addition to analysis of the maximal glutamate concentration change induced by each stimulation train, the temporal dynamics of the stimulation-induced glutamate spike were also analyzed (time to peak response and decay time).

**Figure 2.**
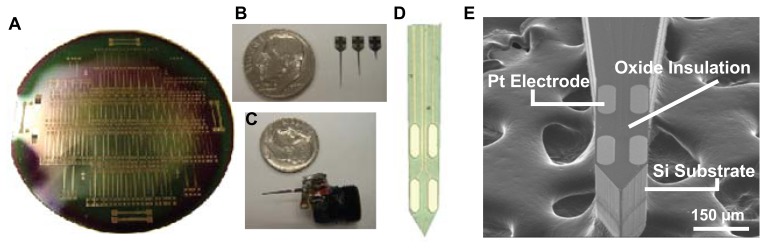
Fabricated silicon wafer-based MEA probes. **(a)** Single 4 in Si wafer with 150 probes. **(b)** Released probes of 3 shaft lengths. **(c)** MEA probe packaged for *in vivo* application. **(d)** tip of MEA probe showing the 4 pt electrodes **(e)** SEM image showing MEA probe tip.

For recording in the awake, freely moving animal connection wires from the MEA and reference electrodes were soldered to gold-plated sockets (Ginder Scientific) and the silicon wafer-based MEA was attached with epoxy to a 9-pin miniature connector (Ginder Scientific) such that all the sockets were encased in the connector. The entire assembly was sealed with epoxy to ensure full insulation and allowed to dry for 1 h prior to implantation. A sensor packaged for freely moving animal experiments is shown in Figure 2C. The two-electrode MEA biosensor, prepared for glutamate detection and pre-calibrated, was unilaterally implanted, along with a contralateral 200 μm-diameter Ag/AgCl reference electrode, into the rat dorsal striatum (AP: +0.7, ML: +2.4, V -7.0) under halothane anesthesia. The connection assembly was anchored to the skull with three stainless steel skull screws along with dental acrylic cement (Bosworth Trim, Stokie, IL). A 48 h recovery period preceded onset of recordings.

Freely moving recordings were conducted in a plexiglass operant chamber (Med Associates, East Fairfield, VT) housed within a sound- and light-resistant shell within a Faraday cage. The recording headstage (Quanteon) consisted of a round miniature connector with 5 connector pins corresponding to the connector housing the implanted biosensor and reference lead. The headstage was tethered to a low torque 12 lead commutator (Airflyte, Bayonne, NJ) mounted on the top of the chamber. Outside of the operant chamber the commutator connected to the FAST-16 potentiostat. This apparatus allowed the animal unrestricted movement within the operant chamber. At test, the headstage was connected to the implanted MEA biosensor in the awake animal and a potential of 0.7 V versus Ag/AgCl was applied. Amperometric data were collected at 80 kHz and averaged over 1 s intervals. The sensor was allowed to equilibrate to baseline for 1 h before experimentation began. After baseline current was attained, the sampling average was lowered to 0.5 s for the majority of the experiment. Mild 1 s tail pinches were administered using stainless steel forceps. All data were plotted as current versus time (GraphPad Prism) and the *in vitro* calibration factor was used to convert current changes to glutamate concentration changes.

## 3. Results and Discussion

### 3.1. Silicon Wafer-Based Platinum Microelectrode Array Glutamate Biosensors

Figure 2 represents the output of the micromachining process. Each 4 inch silicon wafer (Figure 2A) houses 150 MEA probes of varying designs. Figure 2E shows a scanning electron microscope (SEM) image of the MEA tip of a single probe. The probe shafts are 150 μm thick and 120 μm wide. The electrode sites are oval in shape with an approximate width of 40 μm, a length of 100 μm, and a surface area of approximately 4,800 μm^2^. Horizontally paired electrodes are positioned 40 μm apart, with the vertical distance between pairs at 100 μm. Figure 2B shows three probes of varying shaft lengths (2 mm, 6 mm, 9 mm), designed to reach any part of the rat or mouse brain. Figure 2C shows a probe packaged for implantation into the rat brain for awake, freely moving experiments.

Our silicon wafer-based MEA probes are inexpensive to produce and can be fabricated in large quantities with high reproducibility and yield. These MEA probes have superior mechanical strength and are able to withstand implantation into the rodent brain through the dura mater. The probe shaft insulation is robust both *in vitro* and *in vivo*. Of note, the size and shape of each electrode on the MEA as well as the dimensions of the MEA shaft tip itself make these probes ideal for implantation and recording specifically from small regions of the rodent brain such as the NAc. Such specific spatial resolution is necessary for understanding how changes in glutamate may underlie specific behaviors, as activity in adjacent brain regions has been shown to control opposing and dissociable aspects of behavior [22]. The design of our probes is also readily altered based on feedback from *in vivo* experiments. Additionally, the 4 site MEA allows the opportunity for multiple recordings of glutamate from adjacent parts of a given brain region, collection of data from uncoated sites to control for potential changes in endogenous peroxide or, in the future, simultaneous recording of multiple neurotransmitters.

**Figure 3.**
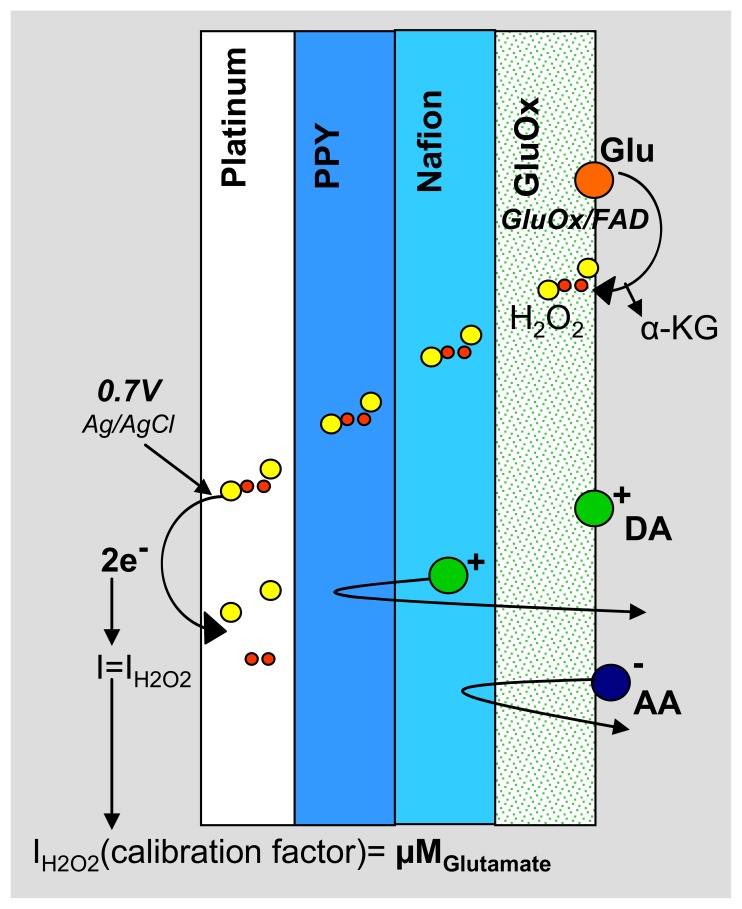
Schematic representation of the coatings on a single electrode on the MEA.

The PPy/Nafion/GluOx-coated electrode is able to amperometrically detect glutamate without interference from AA and DA (Figure 3). GluOx catalyzes the oxidation of glutamate to α-ketaglutarate and H_2_O_2_[14]. The PPy and Nafion layers act as size and charge exclusion membranes, blocking common interferents present in the extracellular fluid, while allowing small hydrogen peroxide molecules to permeate to the electrode surface where an anodic potential is applied [23, 24]. The resulting H_2_O_2_ oxidation current is proportional to the concentration of glutamate present near the electrode surface, thereby acting as the signal output of the sensor.

### 3.2. Sensitive, Selective and Rapid Detection of Glutamate In Vitro

**Figure 4.**
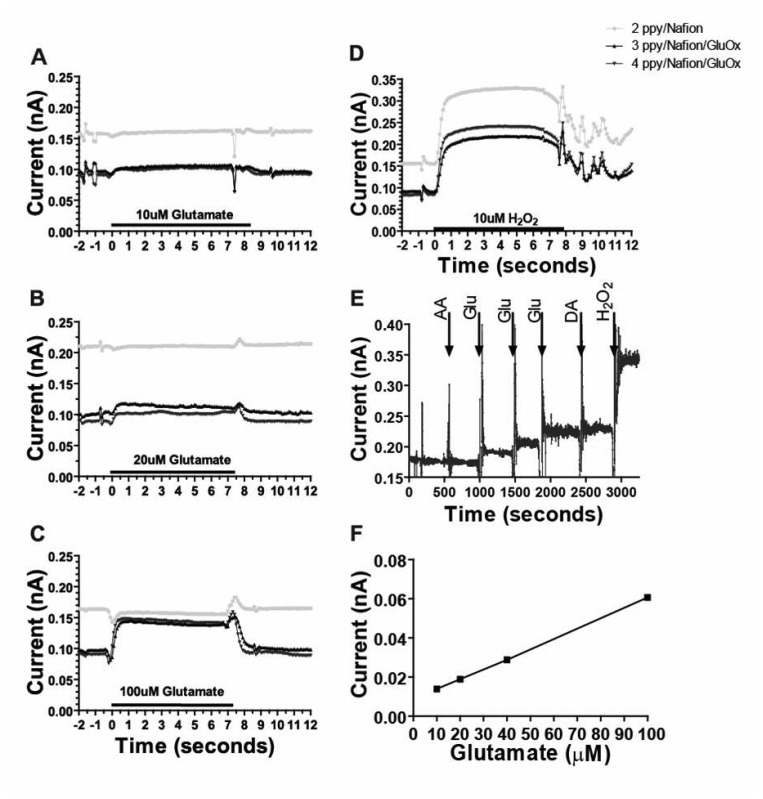
**(a-d)** Representative flow cell calibration data from a MEA coated with PPy/Nafion/GluOx on two electrodes and PPy/Nafion alone on one electrode plotted as current versus time. (Calibration timescale corrected for flow cell dead time). **(e)** Sequential addition of AA (250μM), glutamate (20 μM), DA (5 μM) and H_2_O_2_ (20 μM) in stirred solution. **(f)** Linear *in vitro* current v glutamate concentration relationship.

*In vitro* tests demonstrated that the MEA biosensors coated with PPy/Nafion/GluOx were sensitive to and selective for glutamate *in vitro*. As shown in Figure 4, additions of increasing concentrations of glutamate produced corresponding (Figures 4A, B, C) and linear (Figure 4F) increases in current such that the sensitivity to glutamate was 2.46 +/- 0.48 pA/μM, and the limit of glutamate detection was 0.79 +/- 0.16 μM (11 electrodes on 5 probes) at twice the level of the noise. Given the oxygen-dependent sensing mechanism employed, this linear glutamate calibration curve cannot be assumed accurate for all conditions *in vivo*, especially at very low oxygen concentrations associated with ischemia. Nonetheless, these data, along with work from McMahon and colleagues, indicate these sensors are appropriate for detection of small changes in glutamate concentration *in vivo* under normal physiological conditions [25, 26]. Figure 4 also shows the important lack of response to glutamate at a site on the same probe coated with PPy/Nafion alone, (Figure 4A, B, C), despite this site being sensitive to H_2_O_2_ (Figure 4D). The response to glutamate was rapid; the average time to reach 90% of the maximal current induced by application of glutamate in the flow cell apparatus at electrodes coated with PPy/Nafion/GluOx was 0.8 +/- 0.2 s, (corrected for dead time, 8 electrode sites on 4 probes). This response time is comparable to our previously reported over-oxidized PPy coated sensors [13, 24] and is also similar to the response time to H_2_O_2_ on PPy/Nafion-coated sites devoid of enzyme.

Electroactive interference, in particular from AA and electroactive cations such as DA in the brain, has been a major problem in development of electro-oxidative detection methods for glutamate. Importantly, therefore, PPy/Nafion/GluOx-coated platinum electrodes on the MEA probes were found to be insensitive to interference from DA and AA *in vitro*. Application of 250 μM AA and 5-20 μM DA failed to produce current responses greater than the noise (Figure 4E). Additional tests demonstrated the stability of the interferent exclusion in storage for up to one week after preparation (data not shown), but we routinely implant MEAs within one day of coating and on the same day as calibration. The degree of selectivity for glutamate at our sensors matches that which we previously reported for over-oxidized PPy (without Nafion) on both wire electrodes [13] and ceramic MEAs [24]. However, the combined use of (non-over-oxidized) PPy and Nafion on the current MEAs improved the success rate of obtaining suitably selective sensors without compromising response time.

### 3.3. In Vivo Cortical Stimulation-Evoked Glutamate Release in the Ventral Striatum

**Figure 5.**
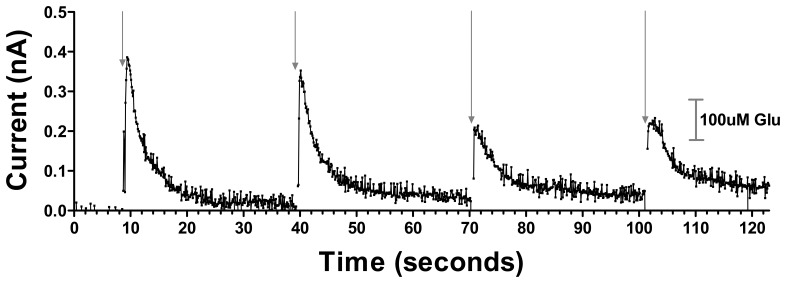
Representative current versus time data showing cortical stimulation evoked current changes corresponding to glutamate concentration changes in the NAc of an anesthetized rat. Each arrow shows the time of 700 μA, 500 Hz 0.5s stimulation delivery. A calibration bar is shown on the right.

The silicon wafer-based MEA glutamate biosensors allow for reliable and near real-time recording of glutamate release in the VS elicited by afferent stimulation of the mPFC (Figure 5). Stimulus trains of 0.5 s in duration (700 μA, 500 Hz) produced an initial current response corresponding to a 326.7 μM glutamate concentration change, which diminished in amplitude approximately 45% with successive stimulations administered 30 s apart, presumably reflecting the depletion of the readily releasable pool of glutamate, supporting the physiological nature of the response.

The concentration changes reported are approximate and uncorrected for differences between diffusion of glutamate in vivo and in solution, potential degradation of sensor activity in vivo, and possible chemical cross-talk between electrode sites due to H_2_O_2_ diffusion. Future studies are required to determine the minimum separation distance between electrode sites to avoid such potential cross-talk. These signals reached 80% of their maximum amplitude within 0.8 s and attained maximal current within 1.3 s. The decay time back to 30% of baseline occurred within 3.5 s. No current response was detected after even the highest stimulation at a subsequently implanted electrode coated with PPy/Nafion but devoid of GluOx in the same location of the same rat, further verifying that the response detected at the PPy/Nafion/GluOx electrode was indeed the result of glutamate concentration changes (data not shown).

These data show the novel ability of our silicon wafer-based MEA glutamate biosensors to detect in near real-time cortically-evoked glutamate release in the NAc of anesthetized rats. Given that the release was detected after neuronal stimulation, it is likely that at least in part our glutamate signal was the result of neuronal glutamate release. Similar stimulated glutamate release has been detected in the VS of freely moving rats using microdialysis [27], but poor temporal resolution of that technique did not offer information on the release dynamics afforded in the current data by the near real-time detection of our biosensors. This study did however provide evidence of the significance of mPFC-driven accumbal glutamate in reward related behavior [27]. Indeed, changes in glutamate release in the VS have been shown to be necessary for both cocaine and heroin seeking behavior [28, 29]. These data provide strong evidence for the utility of these glutamate biosensors for assessing, in specific brain regions, the glutamatergic component of reward related behavior.

### 3.4. Stress-Induced Striatal Glutamate Release in Awake Freely Moving Rat

**Figure 6.**
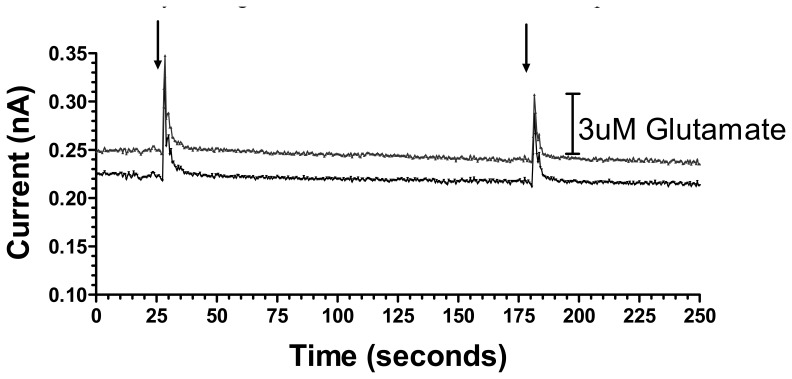
Stress-induced current changes corresponding to glutamate release in the dorsal striatum of a freely-moving rat. Arrows indicate each mild 1 s tail pinch.

As an initial step to demonstrate the utility of these sensors to detect behaviorally relevant rapid glutamate concentration changes in the freely moving rat, a 1 s tail pinch was used as a mild stressor to induce glutamate release in the dorsal striatum. Figure 6 shows representative responses at two PPy/Nafion/GluOx-coated electrode sites of an MEA implanted in the dorsal striatum.

Each tail pinch evoked an immediate increase in the current corresponding to glutamate concentration changes of 2.9 μM and 4.9 μM at the two electrode sites. Not surprisingly, these are much smaller responses than produced by the high intensity, high frequency stimulation described above but, similar to the electrical stimulation data, subsequent tail pinches resulted in approximately a 40% decrease in the glutamate concentration change, in this case perhaps reflecting desensitization of the stress response. Pinch-induced spikes reached on average a maximum current within 1.5 s and decayed to 30% of baseline within 2-3 s of maximal response (Figure 6).

In addition to showing a role for striatal glutamate release in mild stress, these data clearly show the utility of our silicon wafer-based MEA glutamate biosensors for detecting behaviorally relevant rapid glutamate concentration changes in the freely moving animal. Others have reported similar tail-pinch induced glutamate concentration changes of approximately 0.5 μM detected at platinum wire-based glutamate biosensors in the ambulant animal [12]. The current data show increased responsiveness as here a 1 s tail pinch induced a larger increase in glutamate than the 10 s stimulus used in the previous study. This could be due to a number of variables but one factor could be the discrete nature of the recording sites on the MEA relative to the large surface area of the wire electrodes, the latter potentially resulting in averaging out of large spatially resolved changes in extracellular glutamate concentration. Previous work has also shown 5 min tail-pinch induced glutamate release detected on ceramic MEA glutamate biosensors [30]. Indeed, this work shows a more robust glutamate response to this much more robust stressor [30]. The current result provides evidence of the capability of our silicon wafer-based MEA glutamate biosensors to detect relatively minor stress-induced glutamate release in the striatum of the ambulant rat, indicating their potential ability to detect subtle changes in glutamate concentration associated with specific behaviors.

## 4. Conclusions

We report a novel silicon wafer-based MEA glutamate biosensor created with MEMS technologies for sensitive, selective, temporally and spatially precise measurement of glutamate in vivo. PPy/Nafion/GluOx coatings provided effective rejection of the electroactive interferents, AA and DA, along with rapid and sensitive glutamate detection both in vitro and in vivo. The utility of these sensors for measuring electrical stimulation- and behaviorally-evoked glutamate release in vivo was established.
